# Correction: Dearomatization of benzopyrylium triflates with sulfoxonium ylides

**DOI:** 10.1039/d3cc90024j

**Published:** 2023-01-18

**Authors:** Alexandria N. Leveille, Marissa M. Allegrezza, Kalen Laybourn, Anita E. Mattson

**Affiliations:** a Department of Chemistry and Biochemistry, Worcester Polytechnic Institute 100 Institute Road Worcester MA 01609 USA aemattson@wpi.edu

## Abstract

Correction for ‘Dearomatization of benzopyrylium triflates with sulfoxonium ylides’ by Alexandria N. Leveille *et al.*, *Chem. Commun.*, 2022, **58**, 12600–12603, https://doi.org/10.1039/D2CC02023H.

The authors regret that the original article included minor errors in the sulfoxonium ylide structure shown in Schemes 2 and 3, and in Table 4, and in compound **3f** shown in Table 1. The corrected schemes and tables are presented here.

**Scheme 2:**
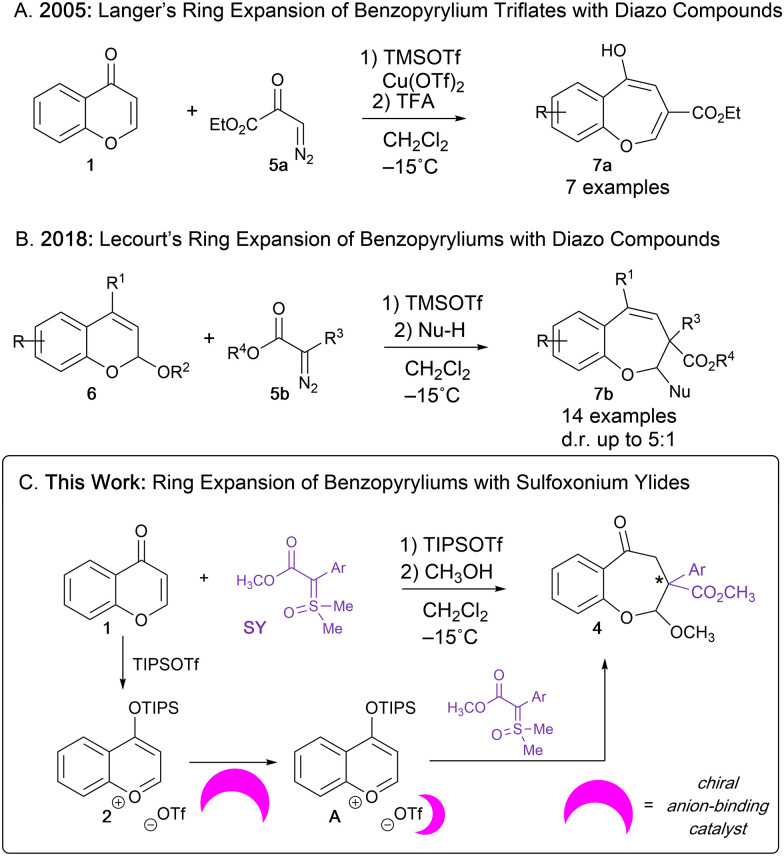
Ring expansion of benzopyrylium ions generated *in situ* to access benzoxepines.

**Scheme 3:**
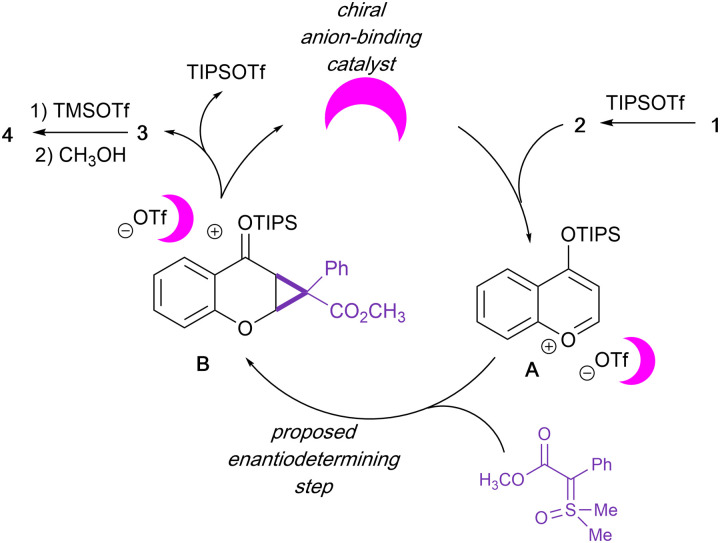
Plausible reaction pathway for enantioselective cyclopropanation and ring-opening sequence.

**Table:** Substrate screen based on sulfoxonium ylides

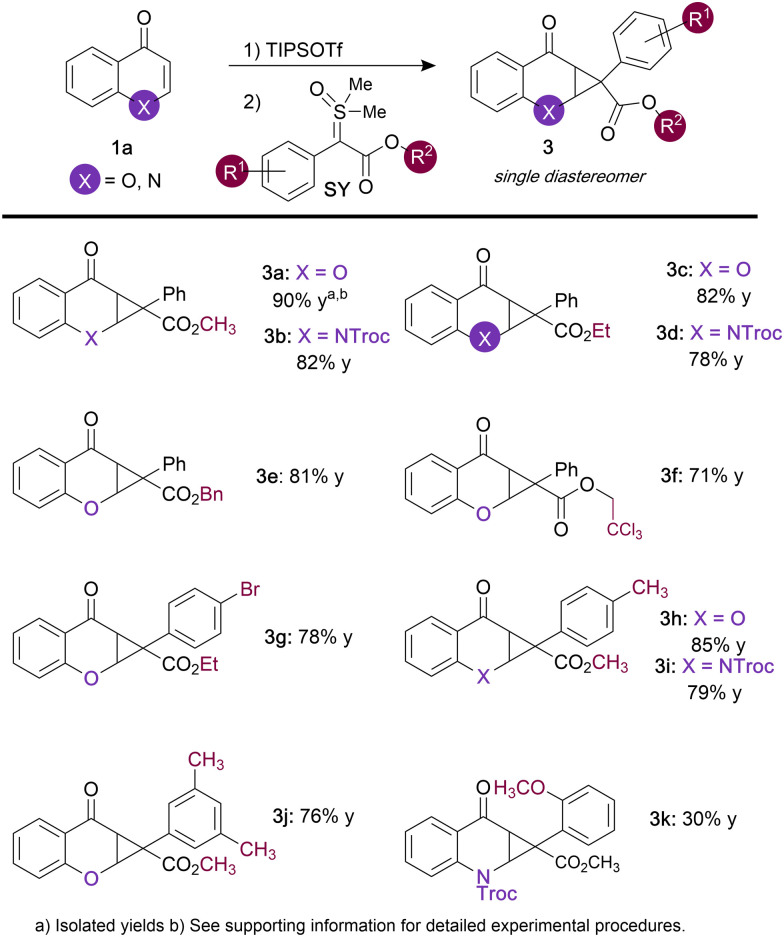

**Table:** Influence of anion-binding catalysts on enantiocontrol

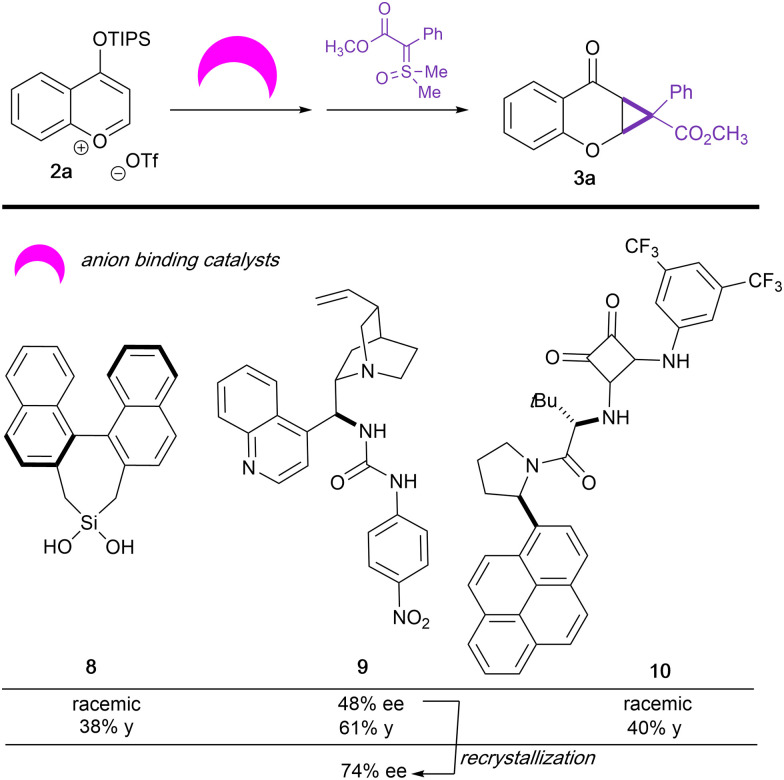

The Royal Society of Chemistry apologises for these errors and any consequent inconvenience to authors and readers.

## Supplementary Material

